# Methylomic signature of current cannabis use in two first-episode psychosis cohorts

**DOI:** 10.1038/s41380-024-02689-0

**Published:** 2024-10-16

**Authors:** Emma L. Dempster, Chloe C. Y. Wong, Joe Burrage, Eilis Hannon, Diego Quattrone, Giulia Trotta, Victoria Rodriguez, Luis Alameda, Edoardo Spinazzola, Giada Tripoli, Isabelle Austin-Zimmerman, Zhikun Li, Charlotte Gayer-Anderson, Tom P. Freeman, Emma C. Johnson, Hannah E. Jongsma, Simona Stilo, Caterina La Cascia, Laura Ferraro, Daniele La Barbera, Antonio Lasalvia, Sarah Tosato, Ilaria Tarricone, Giuseppe D’Andrea, Michela Galatolo, Andrea Tortelli, Maurizio Pompili, Jean-Paul Selten, Lieuwe de Haan, Paulo Rossi Menezes, Cristina M. Del Ben, Jose Luis Santos, Manuel Arrojo, Julio Bobes, Julio Sanjuán, Miguel Bernardo, Celso Arango, Peter B. Jones, Gerome Breen, Valeria Mondelli, Paola Dazzan, Conrad Iyegbe, Evangelos Vassos, Craig Morgan, Diptendu Mukherjee, Jim van Os, Bart Rutten, Michael C. O’Donovan, Pak Sham, Jonathan Mill, Robin Murray, Marta Di Forti

**Affiliations:** 1 Department of Clinical & Biomedical Sciences, University of Exeter Medical School, University of Exeter, Exeter, UK; 2 Department of Social, Genetic and Developmental Psychiatry, Institute of Psychiatry, Psychology and Neuroscience, King’s College London, London, UK; 3 Department of Psychosis Studies, Institute of Psychiatry, Psychology & Neuroscience, King’s College London, London, UK; 4 Department of Health Service and Population Research, Institute of Psychiatry, Psychology and Neuroscience, King’s College London, London, UK; 5 Addiction and Mental Health Group (AIM), Department of Psychology, University of Bath, Bath, UK; 6 Department of Psychiatry, Washington University School of Medicine, St. Louis, MO USA; 7 Centre for Transcultural Psychiatry ‘Veldzicht’, Balkbrug, The Netherlands; 8 Department of Mental Health and Addiction Services, ASP Crotone, Crotone, Italy; 9 Biomedicine, Neuroscience and Advanced Diagnostic Department, Psychiatry Section, University of Palermo, Palermo, Italy; 10 Section of Psychiatry, Department of Neuroscience, Biomedicine and Movement Sciences, University of Verona, Verona, Italy; 11 Department of Medical and Surgical Science, Psychiatry Unit, Alma Mater Studiorum Università di Bologna, Bologna, Italy; 12 Institut Mondor de recherché biomedicale, Creteil, France; 13 Department of Neurosciences, Mental Health and Sensory Organs, Suicide Prevention Center, Sant’Andrea Hospital, Sapienza University of Rome, Rome, Italy; 14 Rivierduinen Institute for Mental Health Care, Leiden, The Netherlands; 15 Early Psychosis Section, Amsterdam UMC, Academic Medical Centre, University of Amsterdam, Meibergdreef 5, 1105 AZ Amsterdam, The Netherlands; 16 Department of Preventive Medicine, Faculdade de Medicina, Universidade of São Paulo, São Paulo, Brazil; 17 Department of Psychiatry, Servicio de Psiquiatría Hospital “Virgen de la Luz”, Cuenca, Spain; 18 Department of Psychiatry, Psychiatric Genetic Group, Instituto de Investigación Sanitaria de Santiago de Compostela, Complejo Hospitalario Universitario de Santiago de Compostela, Santiago, Spain; 19 Department of Medicine, Psychiatry Area, School of Medicine, Universidad de Oviedo, Centro de Investigación Biomédica en Red de Salud Mental (CIBERSAM), Oviedo, Spain; 20 Department of Psychiatry, School of Medicine, Universidad de Valencia, Centro de Investigación Biomédica en Red de Salud Mental (CIBERSAM), Valencia, Spain; 21 Barcelona Clinic Schizophrenia Unit, Neuroscience Institute, Hospital Clinic of Barcelona, University of Barcelona, Institut d’Investigacions Biomèdiques August Pi I Sunyer (IDIBAPS), Biomedical Research Networking Centre in Mental Health (CIBERSAM), Barcelona, Spain; 22 Department of Child and Adolescent Psychiatry, Institute of Psychiatry and Mental Health, Hospital General Universitario Gregorio Marañón, School of Medicine, Universidad Complutense, IiSGM, CIBERSAM, Madrid, Spain; 23 Department of Psychiatry, University of Cambridge, Cambridge, UK; 24 Department of Psychological Medicine, Kings College London, London, UK; 25 Department of Genetics and Genomic Sciences, Icahn School of Medicine at Mount Sinai, New York, NY USA; 26 MRC Centre for Neurodevelopmental Disorders, King’s College London, London, UK; 27 Department of Psychiatry and Neuropsychology, School for Mental Health and Neuroscience, South Limburg Mental Health Research and Teaching Network, Maastricht University Medical Centre, Maastricht, The Netherlands; 28 Department Psychiatry, Brain Centre Rudolf Magnus, Utrecht University Medical Centre, Utrecht, The Netherlands; 29 Division of Psychological Medicine and Clinical Neurosciences, Centre for Neuropsychiatric Genetics and Genomics, Cardiff University, Cardiff, UK; 30 Department of Psychiatry, the University of Hong Kong, Hong Kong, China; 31 Centre for Genomic Sciences, Li KaShing Faculty of Medicine, The University of Hong Kong, Hong Kong, China

**Keywords:** Genetics, Molecular biology

## Abstract

The rising prevalence and legalisation of cannabis worldwide have underscored the need for a comprehensive understanding of its biological impact, particularly on mental health. Epigenetic mechanisms, specifically DNA methylation, have gained increasing recognition as vital factors in the interplay between risk factors and mental health. This study aimed to explore the effects of current cannabis use and high-potency cannabis on DNA methylation in two independent cohorts of individuals experiencing first-episode psychosis (FEP) compared to control subjects. The combined sample consisted of 682 participants (188 current cannabis users and 494 never users). DNA methylation profiles were generated on blood-derived DNA samples using the Illumina DNA methylation array platform. A meta-analysis across cohorts identified one CpG site (cg11669285) in the *CAVIN1* gene that showed differential methylation with current cannabis use, surpassing the array-wide significance threshold, and independent of the tobacco-related epigenetic signature. Furthermore, a CpG site localised in the *MCU* gene (cg11669285) achieved array-wide significance in an analysis of the effect of high-potency (THC = > 10%) current cannabis use. Pathway and regional analyses identified cannabis-related epigenetic variation proximal to genes linked to immune and mitochondrial function, both of which are known to be influenced by cannabinoids. Interestingly, a model including an interaction term between cannabis use and FEP status identified two sites that were significantly associated with current cannabis use with a nominally significant interaction suggesting that FEP status might moderate how cannabis use affects DNA methylation. Overall, these findings contribute to our understanding of the epigenetic impact of current cannabis use and highlight potential molecular pathways affected by cannabis exposure.

## Introduction

The global rise in cannabis consumption has highlighted the need to comprehensively understand its underlying biological effects, particularly those related to mental health. There has been an increase in cannabis use disorders (CUD) worldwide [1] and meta-analyses and neurobiological studies on cannabis use consistently report a dose-response association between heavy cannabis use and increased risk of psychosis [2]. Factors including early adolescence onset, daily use, and the use of high-potency cannabis formulations with high concentrations of Delta-9-tetrahydrocannabinol (THC) are known to be strong predictors of psychotic disorders, reportedly increasing the risk of illness by 2 to 5 times compared to non-users [3]. Moreover, genetic studies have reported a complex and yet-to-be-fully elucidated bidirectional relationship between the genetics of schizophrenia and heavy cannabis use [4–6]. Nevertheless, recent epidemiological evidence suggests that countries with higher prevalence and increased availability of high-potency cannabis have also witnessed elevated incidences of psychotic disorders [7, 8].

Epigenetic mechanisms, particularly DNA methylation, are increasingly recognised as important factors mediating the interplay between risk factors and disease [9]. While several studies have investigated genome-wide DNA methylation changes associated with tobacco smoking, resulting in the development of a reliable DNA methylation based smoking score that distinguishes between current smokers, ex-smokers, and non-smokers [10–12], similar investigations for cannabis use are still in their early stages. Recent studies have started to explore DNA methylation changes associated with lifetime cannabis use [13] and CUD [14], revealing alterations in genes involved in brain development, synaptic function, and mood disorders. Pathway analyses based on cell type-specific DNA methylation changes associated with cannabis use have also implicated pathways regulated by the endocannabinoid system during brain cortical development and pathways involved in DNA repair [15, 16]. Candidate gene studies from peripheral blood have reported changes in DNA methylation associated with heavy cannabis use in genes such as *DRD2, DAT1*, and *COMT*, all of which are involved in dopamine transmission [17–19]. Inconsistencies have been observed regarding changes in the expression of the *CNR1* gene, encoding the Cannabinoid Receptor Gene 1 (CB1), in patients with schizophrenia and cannabis users [20]. The CB1 receptor is the primary target of THC, and its activation modulates inhibitory and excitatory neurotransmission across the central nervous system [21].

Recent studies have also explored the impact of cannabis use on epigenetic age. Epigenetic age reflects an estimation of chronological and/or biological age based on the methylation status of numerous CpG sites across the epigenome [22]. There are numerous “epigenetic clocks” that capture different aspects of biological ageing and recent research suggests that both cannabis use and psychiatric disorders are associated with increased age acceleration (where an individual’s epigenetic age is older than their chronological age) [23]. This has led to the possibility of epigenetic ageing becoming a biomarker for disease susceptibility and lifespan, (in principle) under the influence of environmental exposures such as cannabis.

This study represents the first epigenome-wide association study (EWAS) to explore the impact of current cannabis use, including usage frequency and potency, on DNA methylation. We sought to investigate whether current cannabis use, particularly high-potency types, leaves a signature on DNA methylation and whether this effect is moderated by first-episode psychosis (FEP). Our results highlight the epigenetic impact of current cannabis use and identify potential molecular pathways affected by cannabis exposure.

## Materials and methods

### Sample cohorts

#### GAP study

The GAP study consists of all patients aged 18–65 years who presented with a first episode of psychosis (FEP) to the Lambeth, Southwark and Croydon adult in-patient units of the South London and Maudsley Mental Health National Health Service (NHS) Foundation Trust between December 2005 and October 2008. Clinical diagnosis was validated by administering the Schedules for Clinical Assessment in Neuropsychiatry (SCAN). During the same period, we recruited a healthy control group (*n* = 370) from the local population living in the area served by the Trust, through internet and newspaper advertisements, and distribution of leaflets at train stations, shops, and job centres. Cannabis was not mentioned in these advertisements. Particular attention was directed to attempting to obtain a control sample representative of the catchment area population at risk [24]. Those who agreed to participate were administered the Psychosis Screening Questionnaire, and excluded if they met criteria for a psychotic disorder or reported previous diagnosis or treatment of psychotic illness. Ethical permission was obtained from the Trust and the Institute of Psychiatry research ethics committee. All study participants signed a consent form allowing publication of data originating from the study [24].

Working samples: Participants who had consented to donate blood for epigenetic analyses were stratified to include only frequent cannabis users who were currently using at the time of the blood collection (cannabis use ≥ once a week *n* = 87), 61 of whom reported use of high-potency cannabis products (Skunk-like type of cannabis [24], see more details in the supplementary Methods section) and, for the primary analysis, as the control group we selected those with no history of cannabis use (*n* = 138 non-users).

#### EU-GEI study

The EU-GEI project set out to estimate (a) the incidence of psychosis across 17 sites, and (b) to recruit first-episode psychosis cases and population controls to investigate risk factors for psychotic disorder [25]. First, incidence rates were estimated by identifying all individuals with FEP who presented to mental health services between May 1, 2010, and April 1, 2015, in 17 catchment areas in England, France, the Netherlands, Italy, Spain, and Brazil. As for the GAP study, we stratified the participants with available DNA samples to include only frequent cannabis users who were currently using at the time of the blood collection (cannabis use ≥ once a week *n* = 101), 67 of whom reported regular use of a high-potency cannabis product (THC = > 10% [26]) (details on the high-potency variable are in the supplementary methods) and as the control group, in the primary analysis, we selected individuals with no history of cannabis use (*n* = 356 non-users). Ethical approval was provided by research ethics committees in each site. All study participants consented to allow the publication of data originating from the study [25].

### DNA methylation profiling in blood-derived DNA

DNA was isolated from whole blood using standard phenol/chloroform methods. Genome-wide DNA methylation was profiled in the GAP cohort using the Illumina 450 K DNA methylation array (Illumina Inc) which interrogates >450,000 DNA methylation sites across the genome [27]. The EU-GEI cohort was analysed using the Illumina EPIC DNA methylation array (Illumina Inc), which interrogates >850,000 DNA methylation sites across the genome [28]. After stringent data quality control (see below) the GAP dataset consisted of DNA methylation estimates for 430,660 DNA methylation sites profiled in 225 samples (87 frequent cannabis users & 138 cannabis naive controls). The EU-GEI dataset consisted of DNA methylation estimates for 808,513 DNA methylation sites profiled in 457 samples (101 frequent cannabis users and 356 cannabis naive controls). We also performed a secondary analysis where we stratified current frequent users to those who were currently using a high-potency cannabis product at least once a week (*n* = 128 across both cohorts). To examine the effect of FEP status on our analyses we also set out to determine if there was an interaction between FEP status and cannabis use (FEP cannabis users= 129, cannabis users with no FEP diagnosis = 59: FEP high-potency cannabis users = 96, high-potency cannabis users with no FEP diagnosis =32) (see Table S14 for the breakdown of samples by cohort).

### DNA methylation data pre-processing and quality control

Raw Illumina array data was processed using the *wateRmelon* package as previously described [29]. Our stringent QC pipeline as previously published [30] was followed which includes: checking methylated and unmethylated signal intensities; calculating a bisulfite conversion statistic for each sample; multidimensional scaling of sites on the X and Y chromosomes separately to confirm reported sex; using the *pfilter*function in *wateRmelon* to exclude samples with >1% of probes with a detection *p* value  >  0.05 and probes with >1% of samples with detection *p* value  > 0.05 and the removal of cross-hybridising and SNP probes [29]. The subsequent normalisation of the DNA methylation data was performed using the *dasen* function in either *wateRmelon* or *bigmelon* [29, 30]. These data have been previously published as a component of a large DNA methylation meta-analysis of psychosis [30]. The raw and processed data are available through GEO accession numbers GSE152027, and GSE152026. *Post- hoc* power analyses were performed using the R package pwr [31].

### Identification of differential DNA methylation associated with frequent cannabis use

To identify associations between variable DNA methylation and current regular cannabis use we fitted regression models using the R (version 3.5.2) statistical environment [32] in the two cohorts. To identify DNA methylation sites associated with cannabis use we conducted an EWAS in which DNA methylation at each probe was regressed against cannabis status using a linear regression model where age, sex, experimental batch, sampling centre, tobacco smoking score, psychosis status, to account for mixed ethnicity the first two genetic principle components were included as described by Hannon et al. [30] and derived cell proportions were included as covariates. Cell proportion estimates were derived from DNA methylation data using the Houseman method [33] (Fig. S5). The DNA methylation tobacco smoking score was calculated using the method described by Elliott et al. [34]. To further explore if higher potency cannabis products had a greater effect on the DNA methylome than cannabis per se, we filtered the cannabis cases to include only those that reported frequent smoking of high-potency cannabis [24, 26] and repeated the EWAS analysis including age, sex, experimental batch, tobacco smoking score, the first two genetic Principal Components (PCs), FEP status and derived cell proportions as covariates. To determine the effect of tobacco smoking on the association with cannabis use the *p* value for the tobacco smoking score covariate was also retrieved from the model (see Supplementary tables). To determine whether FEP status moderates the association between cannabis use and DNA methylation, we ran a model incorporating an interaction term (cannabis use * FEP status), using the same covariates described above.

### Meta-analysis of variable DNA methylation associated with current cannabis use

A meta-analysis of the two datasets was then performed using the *metagen* function in the R package *meta* [35] as there are only two data sets a fixed-effect model was used, using the effect sizes and standard errors from each cohort to calculate weighted pooled estimates and test for significance. Probes were limited to those present in both cohorts (*n* = 399,943). A stringent significance threshold (*p* < 2.4 × 10 − 7) was used that was derived through permutation testing [36]. Pathway analyses were subsequently performed on Differentially Methylated Positions (DMPs) (*p* < 0.001) using the *methylglm* function within the *methylGSA* package developed by Ren and Kuan [37] using the default parameters. *methylglm* adjusts for the number of DNA methylation sites per gene in the logistic regression model. To identify differentially methylated regions (DMRs), we identified spatially correlated *p* values in our meta-analysed results using the Python module *comb-p* [38] to group sequential DMPs (*p* value < 0.001) at a maximum distance of 500 bp. DMR *p* values were corrected for multiple tests using Šidák correction. To determine if FEP status moderated the association between our measures of cannabis use and changes in DNA methylation we ran separate models including an interaction term between (1) current cannabis and FEP status and (2) high-potency current cannabis use and FEP status. To combine the results from the two cohorts for the interaction model, we meta-analysed both the main effect *p* value and the interaction *p* value from both cohorts.

### Epigenetic age acceleration

The estimated “DNA methylation age” for each sample was calculated using four different epigenetic clocks. These included two chronological age predictors, Horvath [39] and Hannum [40] along with two second-generation clocks, PhenoAge, a biomarker of advanced biological aging [41] and the DNA-methylation biomarker of Pace of Aging (DunedinPACE (POA) [42]). To calculate age acceleration DNA methylation age was regressed on chronological age, residuals of these models were then used as the age acceleration measure for Horvath [39], Hannum [40], PhenoAge [41] epigenetic clocks. Linear models were used to determine if age acceleration measures were associated with cannabis use while controlling for age, sex, experimental batch, tobacco smoking score, genetic PCs, FEP status and derived cell proportions as covariates.

## Results

### Overview of experimental strategy and cohort demographics

We quantified genome-wide DNA methylation across two first episode psychosis cohorts (GAP and EU-GEI) using the Illumina Infinium HumanMethylation450 BeadChip (450 K array) and the Illumina EPIC DNA methylation BeadChip (EPIC array) (Illumina Inc., San Diego, CA, USA) respectively (see Materials and Methods). In total, data from 682 samples passed stringent QC metrics and were used for analysis. Full demographic information on the final cohort can be found in Table 1. We found no differences in estimated cell composition between current cannabis users and controls while controlling for tobacco smoking score, age, psychosis status and sex (Figs. S1 & S2; Table S11).

**Table 1:** Characteristics of the samples profiled in this study.

		GAP		EU-GEI		Total
		Current cannabis users ≥ once a week	Cannabis never users	Current cannabis users ≥ once a week	Cannabis never users	
Number		87	138	101	356	682
Female		16 (18%)	68 (49%)	12 (12%)	214 (60%)	45%
Mean Age years ±STD		31.5 ± 7.45	35.64 ± 10.99	27.8 ± 8.66	39.4 ± 13.8	33.89 ± 13.14
Mean age at first cannabis use (years ± STD)		16 ± 4.3	/	16.5 ± 5.2	/	16.3 ± 5.35
Age range		17–50	16–72	18–57	18–64	16–72
FEP Diagnosis	Male	51	36	62	27	176
	Female	9	42	7	5	63
Ethnicity	White European	41	45	61	239	386
	Black African	14	37	8	30	89
	Other	32	56	27	69	170
Early onset of cannabis use (age ≤14)		28	/	34	/	62

Our analyses focussed on identifying differentially methylated positions (DMPs) and differentially methylated regions (DMRs) associated with current frequent cannabis use (see Fig. S3 for a directed acyclic graph (DAG) illustrating the analysis design). *Post hoc* power calculations demonstrate we are adequately powered for the main EWAS analysis. For an average probe in our dataset with a sample size of 188 cases and at least the same number of controls we have >80% power to detect a 2% difference in DNA methylation. However, if we stratify to cannabis users with an FEP diagnosis the power to detect a 2% difference in DNA methylation is reduced, for example with a sample size consisting of 129 cases (cannabis+FEP) and the same number of controls we have 45% power to detect a 2% difference in DNA methylation.

### Methylomic differences between frequent current cannabis use and never–users

**Fig. 1:**
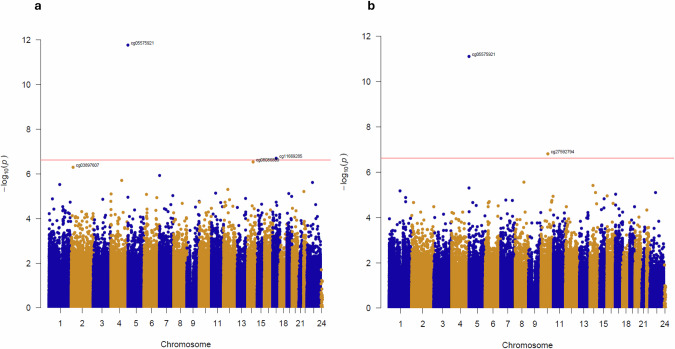
Differentially methylated positions (DMPs) identified in a meta-analysis of current frequent cannabis use and current frequent high-potency cannabis across two cohorts. **a** Manhattan plot highlighting significant DMPs associated with current cannabis use from a comprehensive EWAS meta-analysis of two datasets (total *n* = 682 individuals). In total, 2 DMPs associated with cannabis use were identified at an experiment-wide significance level. The *x*-axis depicts chromosomes 1–22 and the *y*-axis the significance level (−log10(p)) for each DNA methylation site tested. The horizontal red line represents the experiment-wide significance level (*p* < 2.4E-07). Probe annotations are given for the top-ranked DMPs and the list of results is in Table 2 & Table S1. **b** Manhattan plot highlighting significant DMPs associated with current high-potency cannabis use (total *n* = 622 individuals). In total 2 DMPs associated with cannabis use were identified at an experiment-wide significance level. A list of results is given in Table 3 & Table S4.

Our first analyses focussed on identifying DNA methylation differences between frequent current cannabis users (*n* = 188) and never users (*n* = 494) (Fig. 1a). Quantile-quantile plots for the analyses are shown in Fig. S4, highlighting little evidence of systematic *p* value inflation. The 10 top-ranked cannabis-associated DMPs from the meta-analysis are listed in Table 2, with more extensive results and results for the individual cohorts in Tables S1–S3 and Fig. S5. A secondary analysis focused on those individuals who were frequent users of high-potency cannabis (*n* = 128) and never users (*n* = 494) (Fig. 1b). The 10 top-ranked high-THC-associated DMPs from the meta-analysis are listed in Table 3, with more extensive results and results for the individual cohorts in Tables S4–S9.

**Table 2:** Top 10 probes from the current cannabis users EWAS meta-analysis.

Probe	Effect Size	*p* value	CHR	Genomic Position (hg19) (bp)	UCSC RefGene	GREAT gene annotation [73] (with distance from TSS)
cg05575921	−3.75	1.68E-12	5	373378	AHRR	AHRR ( + 69087), C5orf55 ( + 69879)
cg11669285	−2.57	1.93E-07	17	40558061	PTRF/CAVIN1	STAT3 (−17549), PTRF ( + 17276)
cg08086809	−0.76	2.85E-07	14	82000312	SEL1L	SEL1L (−108)
cg03897607	1.22	5.05E-07	2	18768419	NT5C1B	NT5C1B-RDH14 ( + 2426), KCNS3 ( + 708475)
cg20777796	−1.05	1.17E-06	7	1980117	MAD1L1	ELFN1 ( + 231320), MAD1L1 ( + 292465)
cg18578898	−1.34	1.97E-06	4	122086698	TNIP3	NDNF (−93026), QRFPR ( + 215482)
cg26561773	3.09	2.34E-06	X	65259390	VSIG4	VSIG4 ( + 576)
cg14915165	−0.54	2.90E-06	1	118471929	WDR3;GDAP2	WDR3 (−442), GDAP2 ( + 372)
cg23063905	1.34	4.92E-06	12	48129486	RAPGEF3	ENDOU (−10132), RAPGEF3 ( + 23402)
cg10585872	1.09	5.94E-06	22	19647281		CLDN5 (−134422), SEPT5 (−54705)

*TSS* Transcription Start Site, *bp* base pair.

**Table 3:** Top 10 probes from the high-potency current cannabis users EWAS meta-analysis.

Probe	Effect Size	*p* value	CHR	Genomic Position (hg19) (bp)	UCSC RefGene	GREAT gene annotation [73] (with distance from TSS)
cg05575921	−4.09387	7.56E-12	5	373378	AHRR	AHRR ( + 69087), C5orf55 (+69879)
cg27592794	−1.88354	1.54E-07	10	74454766	MCU	OIT3 (−198572), MCU ( + 2878)
cg21333674	−4.59295	2.71E-06	8	96705765		GDF6 ( + 467254), PLEKHF2 ( + 559817)
cg01820754	−1.17263	3.86E-06	14	53418577	FERMT2	FERMT2 (−763)
cg26703534	−2.15739	4.95E-06	5	377358	AHRR	C5orf55 (+65899), AHRR ( + 73067)
cg14915165	−0.60354	6.47E-06	1	118471929	WDR3;GDAP2	WDR3 (−442), GDAP2 ( + 372)
cg08086809	−0.78078	7.85E-06	14	82000312	SEL1L	SEL1L (−108)
cg26561773	3.382006	7.93E-06	X	65259390	VSIG4	VSIG4 ( + 576)
cg00715241	−0.55651	9.46E-06	17	4891081	CAMTA2	CAMTA2 (−417)
cg04184273	2.773642	1.09E-05	16	758683		METRN (−6489), STUB1 ( + 28569)

*TSS* Transcription Start Site, *bp* base pair.

Interestingly the top probe (cg05575921) in the primary and secondary analyses maps to the *AHHR* gene, which is robustly associated with the tobacco smoking score, despite including the methylation derived smoking score in the model as a covariate (cannabis current use effect size = −3.75, *p* = 1.68E-12). Other probes which have not been previously associated with tobacco smoking also reached our array-wide significance threshold (*p* < 2.4 × 10 − 07). Probe cg11669285, which maps to intron 1 of the *CAVIN1/PTRF* gene, was significantly hypomethylated in the current cannabis users (effect size = −2.57, *p* = 1.93E-07) and probe cg27592794, which is situated in intron 1 of the mitochondrial calcium uniporter gene (*MCU*) that mediates calcium uptake into mitochondria, was hypomethylated in high-potency current users (effect size = −1.88, *p* = 1.53E-07).

**Fig. 2:**
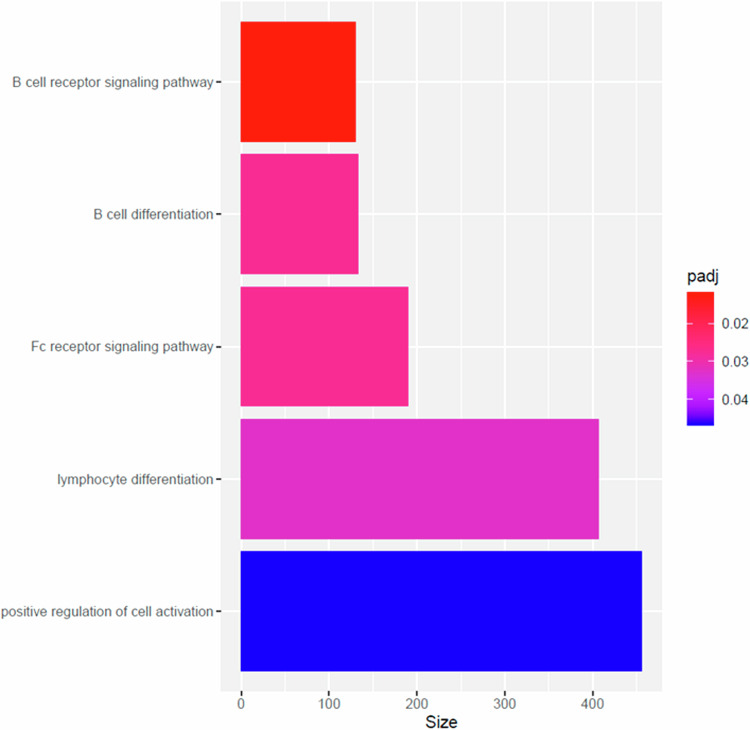
Pathway analyses revealed significant enrichment of biological pathways related to immune processes associated with current cannabis use. Pathway analyses were performed on DMPs from the meta-analysis using the methylGSA package (see Methods). A significant enrichment of biological pathways involved in immune processes such as lymphocyte differentiation (padj=0.032) and B cell receptor signalling (padj=0.012) was observed.

Pathway analyses identified a significant enrichment of biological pathways involved in immune processes such as lymphocyte differentiation (padj=0.032) and B cell receptor signalling (padj=0.011) in the analysis of frequent cannabis and never users (Fig. 2 and Table S13). The high-potency analysis did not identify any pathways after correction for multiple testing (Fig. S6).

**Fig. 3:**
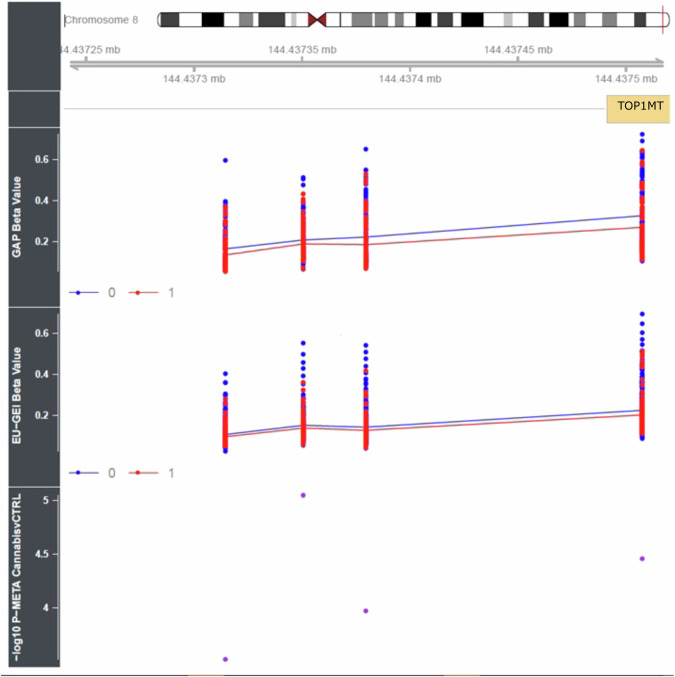
Differential DNA methylation in the promoter region of *TOP1MT* is associated with current frequent cannabis use. Using comb-p [38] we identified regions of differential DNA methylation associated with current cannabis use including a differentially methylated region (DMR) comprising four probes located upstream of the *TOP1MT* gene (Sidak corrected *p* value = 3.88E-07). The figure depicts the specific probes exhibiting differential methylation patterns in both cohorts, with the meta-analysis *p* value plotted in the bottom panel. The beta value corresponds to the DNA methylation level ranging from 0 (unmethylated) to 1 (methylated). Groups are labelled as either 0 (control) or 1 (current cannabis user).

We next used *comb-p* [38] to identify spatially correlated regions of differential DNA methylation significantly associated with cannabis use (Šidák-corrected *P* < 0.05, number of probes ≥ 2) finding a DMR upstream of *TOP1MT*, which encodes a mitochondrial DNA topoisomerase that plays a role in the modification of DNA topology, associated with current cannabis use (chr8:144437314-144437508, Sidak-corrected *p* value = 3.88E-07) (see Fig. 3 with full results in Table S12).

### DNA methylation and cannabis use in first episode psychosis (FEP)

From the interaction analyses, we identified four probes that exhibited a significant main effect (association between the measures of cannabis use and DNA methylation) (*p* < 2.4 × 10 − 07) and a nominally significant interaction term (See Tables S7 & S8 and Fig. S7) indicating that FEP status moderates the association between cannabis and DNA methylation complicating the interpretation of the main effect. In the current cannabis use interaction analysis the probe cg27138281 located in the gene *ME1* (a cytosolic cell metabolism gene) showed a significant main effect (*p* = 3.62E-08) and a nominally significant interaction term (*p* = 3.25E-05). In the high-potency analysis, the probe cg14082739 located in the gene *NEK6* (a protein kinase involved in mitotic cell cycle progression) showed a significant main effect (*p* = 1.47E-07) and a nominally significant interaction term (*p* = 0.0002). There were also two genes with multiple associated probes including *SH3BP4* and *ECHDC3*. *SH3BP4* is involved in endocytic functions and a deletion of this gene has been linked to autism and intellectual disability [43]. *ECHDC3* is predicted to be active in the mitochondrion, which is involved in fatty acid biosynthesis and lipid metabolism. Rare DNA methylation alterations within *ECHDC3* have been previously identified in schizophrenia [44].

### Epigenetic age acceleration and cannabis

We found no evidence for accelerated epigenetic age associated with current cannabis use when controlling for relevant confounders using four independent epigenetic clocks (Figs. S8 & S9, Table S9). Looking specifically at cannabis users with FEP, we saw no evidence of age acceleration with cannabis use.

## Discussion

This study investigated the effect of current cannabis and high-potency cannabis use on DNA methylation in two independent first-episode psychosis case-control cohorts. The majority of the current cannabis users in both our samples used high-potency types (THC = > 10%) more than once a week and they had first used cannabis on average at age 16 years old [26–28]. We identified DMPs associated with the current use of cannabis and high-potency cannabis that was independent of the well-established tobacco smoking episignature. We also identified one DMR that was associated with current cannabis use. In the secondary analyses we included an interaction term to investigate if the cannabis DNA methylation signature is influenced by first-episode psychosis (FEP) status. We identified probes that had a significant main effect and a nominally significant interaction term which suggests a moderating role of FEP status on the cannabis epigenetic signature for certain probes. These results require further replication given the relatively small number of FEP cannabis users and non-users included in the study, but provide insight into the epigenetic impact of cannabis use and potential molecular pathways affected by cannabis exposure.

In our primary analysis, we identified sites annotated to genes associated with mitochondrial functions that were differentially methylated in individuals currently using cannabis regularly. A DMP located in intron 1 of *CAVIN1 (*also known as *PTRF)* was found to be hypomethylated in current cannabis users CAVIN1 is a cytoplasmic protein involved in the formation and function of caveola and has been implicated in mitochondria bioenergetics [45, 46]. In the high-potency analysis, the top DMP was located in intron 1 of the mitochondrial calcium uniporter (*MCU*). Research in astrocytes has found MCU activity is specifically involved in mitochondria CB1 receptor (mtCB1) activation resulting in mitochondrial calcium uptake [47]. Subsequent DMR analyses identified a significant hypomethylated region associated with current cannabis use located in the promoter of *TOP1MT* which encodes a mitochondrial DNA topoisomerase that is important for the regulation of the mitochondrial genome. Interestingly the expression of this gene was found to be significantly upregulated by the demethylating agent 5Aza suggesting that DNA methylation of this gene exerts transcriptional control [48]. These results suggest that current regular cannabis use is associated with hypomethylation in genes involved in mitochondrial function, which could be related to cannabinoid-activated mtCB1 receptor signalling. In support of these findings, a recent RNAseq analysis of human-induced pluripotent stem cells (hiPSCs) exposed to acute or chronic THC exposure identified differential expression in a subset of genes involved in mitochondria function including one of the genes identified in this study, *CAVIN1/PTRF* [49].

Endocannabinoids and cannabinoids bind to two different receptors (Cannabinoid 1 and Cannabinoid 2) which are widespread throughout the body. Cannabinoid 1 receptors (CB1) are predominantly expressed in the central nervous system [50]. Recently it has been established that CB1 receptors are present at the membranes of mitochondrial (mtCB_1_) where they regulate cellular respiration and energy production in different tissues including the brain [51, 52]. There is increasing evidence that cannabinoids modulate mitochondrial processes including, the regulation of intracellular calcium levels [53], activation of the intrinsic apoptotic pathway, impairment of the electron transport chain activity, and disruption of mitochondrial respiration [52]. Further, in mice, cannabinoid-activated mtCB1 receptor signalling has been found to regulate behaviour by controlling astroglial glucose metabolism [54] and cognitive function [55]. Further research should investigate the role cannabis has on mitochondrial function, with a particular focus on tissues with high energy demands, such as the brain where cannabis exerts its psychotropic effects. Both active components of cannabis, THC and CBD, independently influence mitochondrial function and maintenance, which is particularly evident in several neural cell types [54, 55]. Cannabis mediated mitochondrial impairment may affect bioenergetics in the brain affecting critical neuronal processes that could lead to behavioural abnormalities [56]. We postulate that such deficiencies in mitochondrial function could explain some of the sequelae of long-term cannabis use, including elevated risk of psychosis [57] which has been associated with defects in mitochondria function [56]. Pathway analysis also highlighted the enrichment of biological pathways involved in immune processes. Cannabis is known to exhibit immunomodulatory properties and many immune cells express endocannabinoid receptors [58]. Both active components of cannabis have shown to have immunosuppressive properties including reducing the production of pro-inflammatory cytokines and decreasing the activity of certain immune cells, such as T cells and natural killer cells [59].

Despite the limited sample size and the higher levels of cannabis smoking in this group (Table S14), the analysis exploring the interaction between FEP status and cannabis use identified probes that indicate that FEP status may moderate some of the effects of cannabis on DNA methylation. Specifically, in the high-potency analysis, we identified two sites annotated to the gene *ECHDC3*, which is a predicated mitochondrial gene. It is plausible that factors associated with the onset of psychosis such as stress (our cases were all undergoing their first episode of psychosis), hospital admission, starting of treatment, or a differential vulnerability to cannabis exposure in individuals predisposed to psychosis might explain why FEP-status changes the impact of cannabis on the epigenome.

Contrary to previous reports we found no evidence of epigenetic age acceleration in current cannabis and high-potency cannabis users using four different epigenetic clocks. Stratifying the analysis to those with a FEP diagnosis we found no evidence of age acceleration with cannabis use in this group using four different epigenetic clocks including two that reportedly capture biological age more accurately (PhenoAge and PoA). In a longitudinal study conducted by Allen et al. [60], age acceleration, as measured by PoA, was identified in individuals who used cannabis. However, the authors concluded that this association was mediated by a specific site in the *AHHR* gene, known to be linked to tobacco inhalation. This suggests that the observed age acceleration in their population was primarily attributed to the well-established effects of tobacco smoking [61] rather than cannabis use. In our study, we accounted for tobacco smoking as a confounding factor using an established DNA methylation based tobacco smoking score [34] when calculating epigenetic age which may explain the difference in results.

The results of this study should be considered within the context of important limitations. First, it should be noted that the cannabis variables in both cohorts are based on self-report. Nevertheless, in the GAP samples self-report current use was validated in a random subset of participants by urine drug test showing a high inter-rater reliability [24]. The participants reported the street name of the cannabis used, which we were able to categorise in low and high-potency according to official sources available to both studies [26, 62]. A study by Freeman et al. [63] compared self-reported details of the type and potency of the cannabis participants used with (1) THC analysis from the cannabis samples participants provided and (2) their THC blood levels. Their findings showed that cannabis users are reliable in estimating the potency of the cannabis they use even when bought from the illegal market, as for the majority of our study participants. We had official government data on the expected THC content in the different types of cannabis available for illegal and legal use for all the countries our participants come [62, 64–71]. Moreover, these estimates of cannabis potency have been shown, in published epidemiological studies [26–28] to impact on risk of psychosis differentially. Whilst this provided an indication of the potency of cannabis used, the duration of the current use and the precise percentages of THC and CBD they were exposed to could not be precisely estimated. These factors have the potential to influence epigenetic markers and measures of age acceleration. Nevertheless, we successfully stratified users based on their self-reported use of high-potency cannabis, and the findings revealed significant differentially methylated positions (DMPs) even with a smaller sample size. These results suggest that considering the potency of cannabis may be relevant for future research.

Second, although our meta-analysis of EWAS results from two independent cohorts increased our power to detect cannabis-related changes in DNA methylation, we did sacrifice the inclusion of platform-specific information that could have had biological significance given the use of different versions of the Illumina array in each sample cohort.

Third, whilst our secondary analysis that tested for an interaction with FEP disease status suggests that at some DNA methylation sites, FEP status moderates the impact of cannabis use on DNA methylation, the limited sample size and the relatively small numbers of cannabis users without a diagnosis of FEP, warrant future studies on larger sample sizes.

Finally, we may not have fully accounted for the effect of tobacco smoking on the epigenome. As the majority of users in our cohort used cannabis with tobacco (85%) and tobacco-related DNA methylation patterns are highly pronounced in the blood methylome, identifying a distinct cannabis signal becomes challenging. While we did control for tobacco use using the DNA methylation-derived smoking score, we still observed sites associated with cannabis use that were also associated with tobacco smoking e.g., probes located in *AHRR*. Although we attempted to control for confounding in our analyses, it is also possible that the cannabis signal itself is amplifying the smoking signal via the actual combustion of cannabis itself or by the actions of other metabolites. Nevertheless, co-use with tobacco remains the most common mode of cannabis consumption in Europe both among patients with psychosis as well as in the general population. Therefore, as well as separating the effect of the two substances it is important to consider the joint effect, which is more likely to represent the real-world situation. Interestingly, a recent EWAS on PTSD also identified significant alterations in DNA methylation in the *AHRR* gene even in non-smokers suggesting that other factors could be contributing to the association of *AHRR* differential methylation and tobacco smoking [72].

In conclusion, this study identified a DNA methylation blood-based signature of current cannabis use and its potency and suggests there is a moderating role of FEP disease status on the epigenetic signature. Importantly, our findings point to cannabis-related gene regulatory changes in genes related to mitochondrial and immune function. These are biological processes known to be influenced by cannabinoids and have an important role in neurodevelopment throughout the life span including in adolescence when cannabis use is most likely to begin. Future research should further explore the interplay between frequent use of high-potency cannabis and epigenetic changes in biological pathways that can lead to a better understanding of how cannabis affects brain function. This is particularly important at times of increased availability of high-potency cannabis varieties across the world.

## Supplementary information

Supplementary Figures

Supplementary Methods

Supplementary Tables

## Data Availability

1. The datasets analysed during the current study are available in the GEO repository, The raw and processed data are available through GEO accession numbers GSE152027, and GSE152026. 2. Extended results tables for the EWAS analyses are available on the figShare repository 10.6084/m9.figshare.26003827.v1. 3. All data generated or analysed during this study are included in this published article [and its supplementary information files].

## Code availability

Code for the QC of the methylation data is located at: https://github.com/ejh243/SCZEWAS/tree/master/Phase2. Sample scripts for the EWAS and meta-analyses are located at: https://github.com/eld211/CanEWAS.
