# Supplementary material for: Methylomic signature of current cannabis use in two first-episode psychosis cohorts
Source: Mol Psychiatry. 2024 Oct 16;30(4):1277–86. doi: 10.1038/s41380-024-02689-0 (PMC11919776; doi:10.1038/s41380-024-02689-0)
Supplement: Supplementary file 1 — Supplementary Figures [file 41380_2024_2689_MOESM1_ESM.docx]

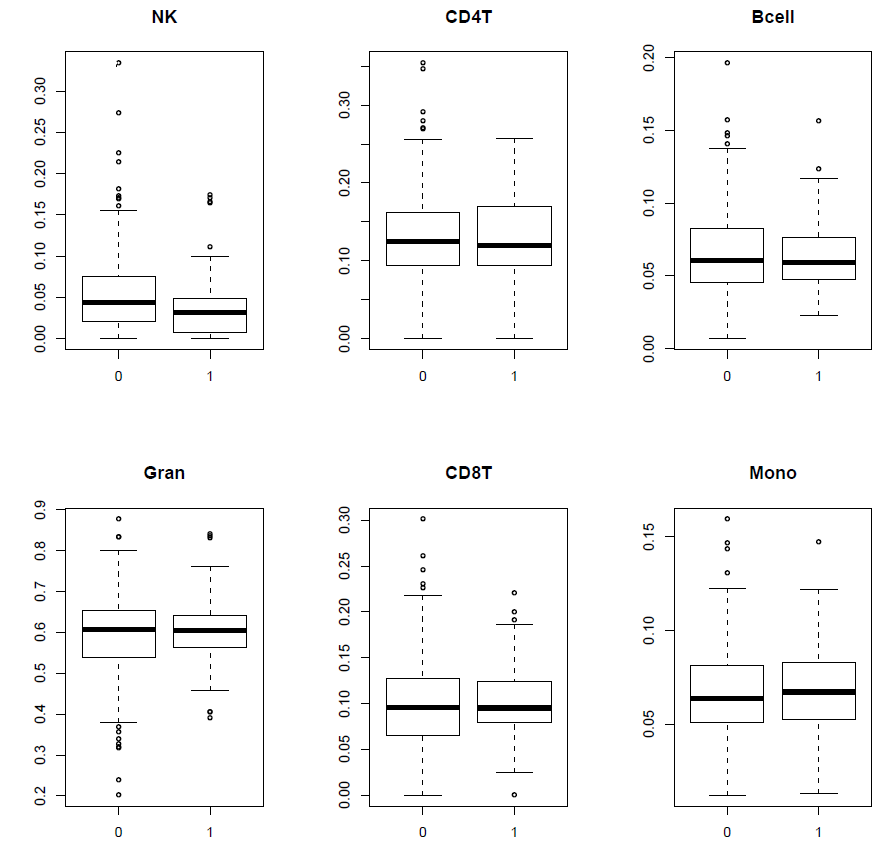


**Figure S1: Investigation of the association between DNA methylation estimated cell composition and current cannabis use in the EU-GEI cohort**. (0= controls 1=cannabis current user)


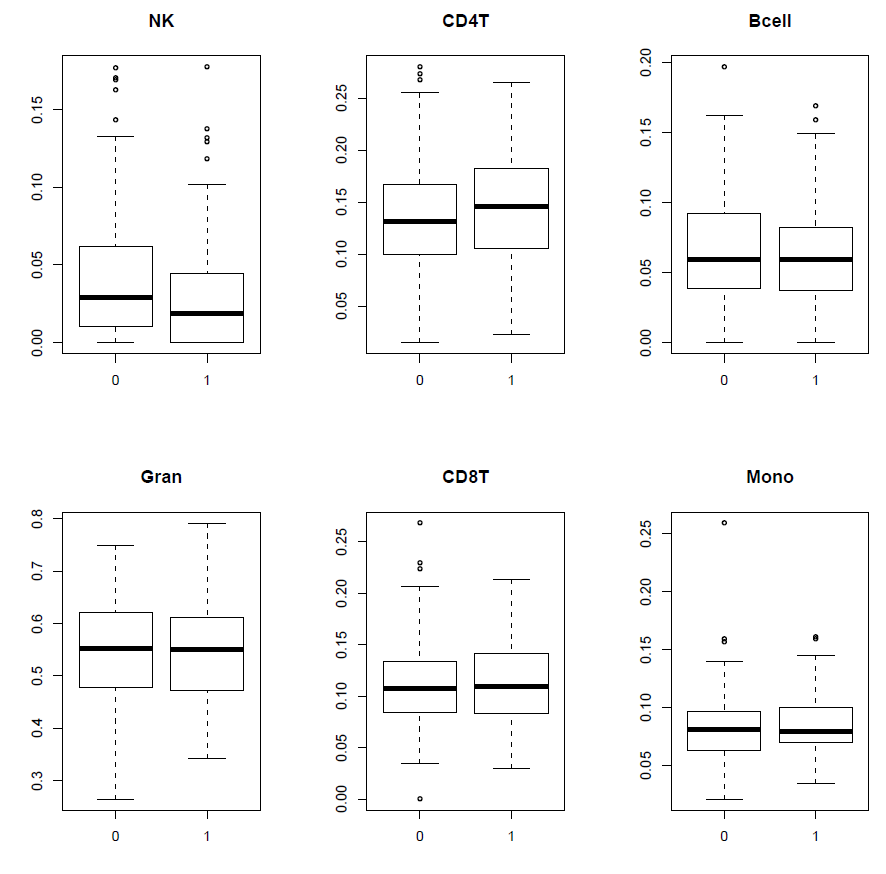


**Figure S2: Investigation of the association between DNA methylation estimated cell composition and current cannabis use in the GAP cohort.** (0= controls 1=cannabis current user)


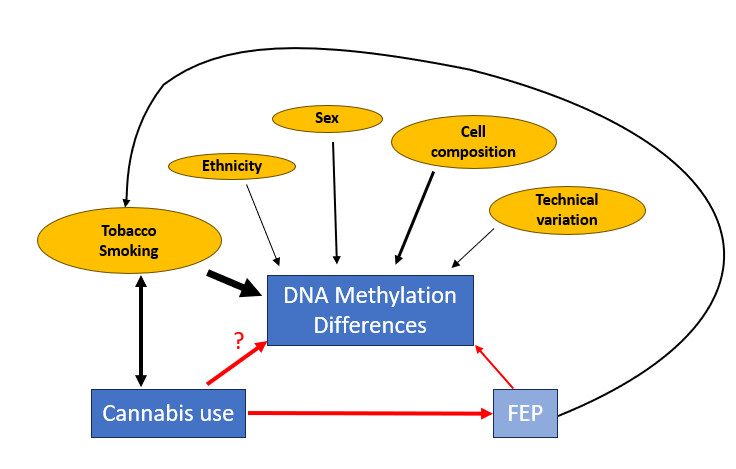


**Figure S3: Directed Acyclic Graph (DAG) – graphically illustrating the analyses performed in this study.** The main analysis is to determine if there is a DNA methylation signature of current cannabis use that is independent of the well-established tobacco smoking signature. The second outcome of our analysis was to determine if having a diagnosis of FEP modified the relationship between cannabis use and DNA methylation using an interaction model.


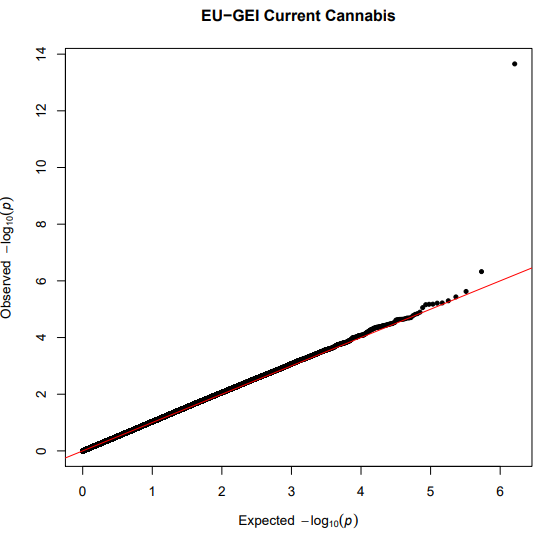

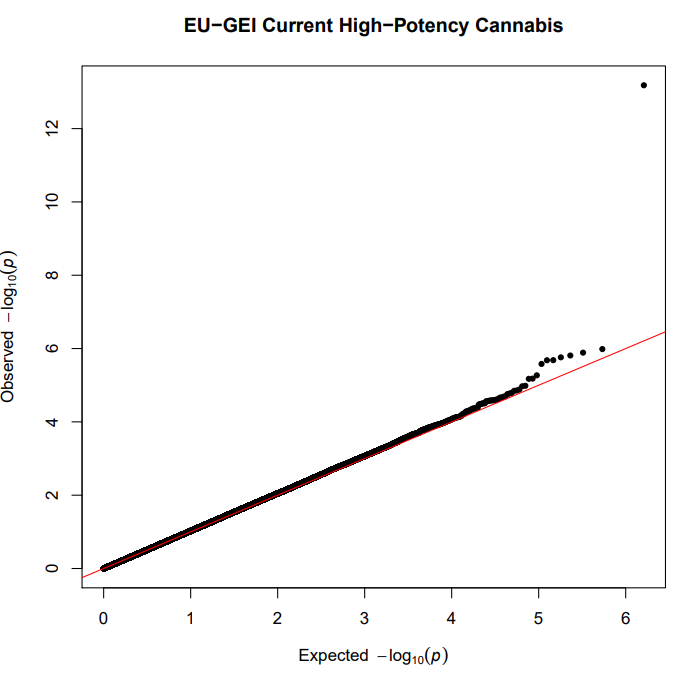


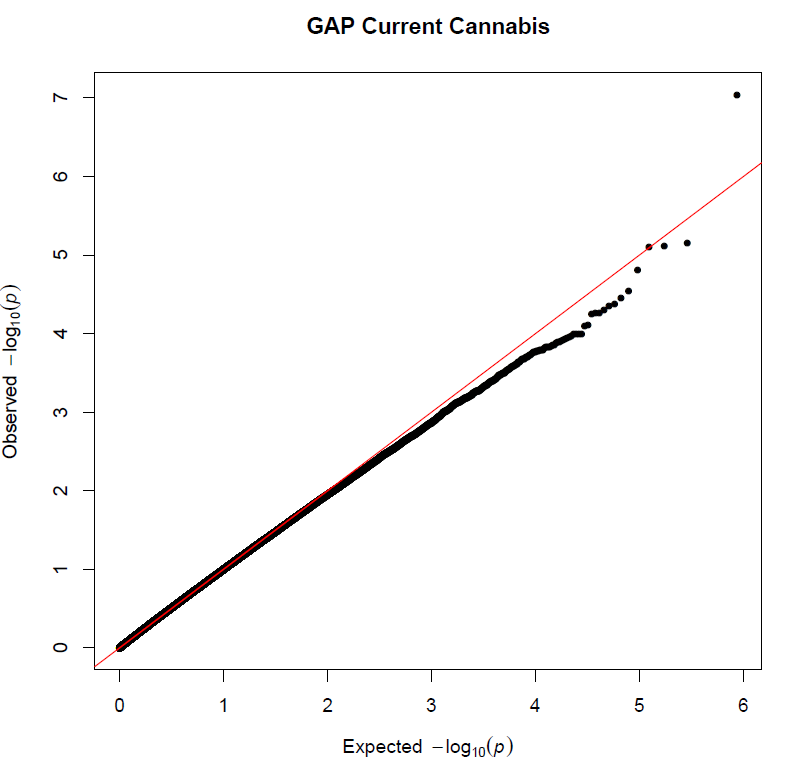

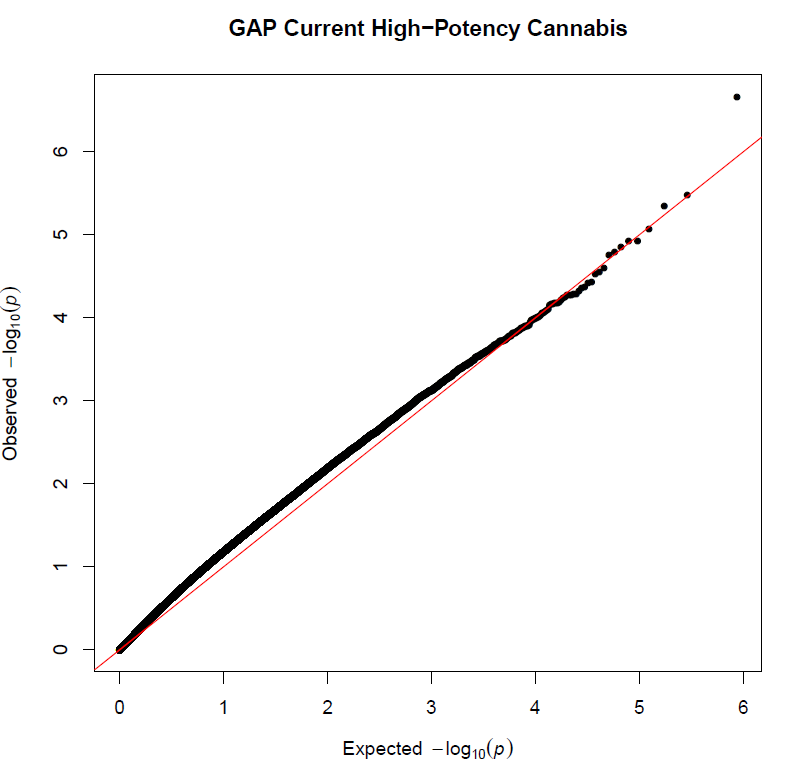


**Figure S4. QQ-plots for each analysis in the two different cohorts**: No evidence of p-value inflation was observed.


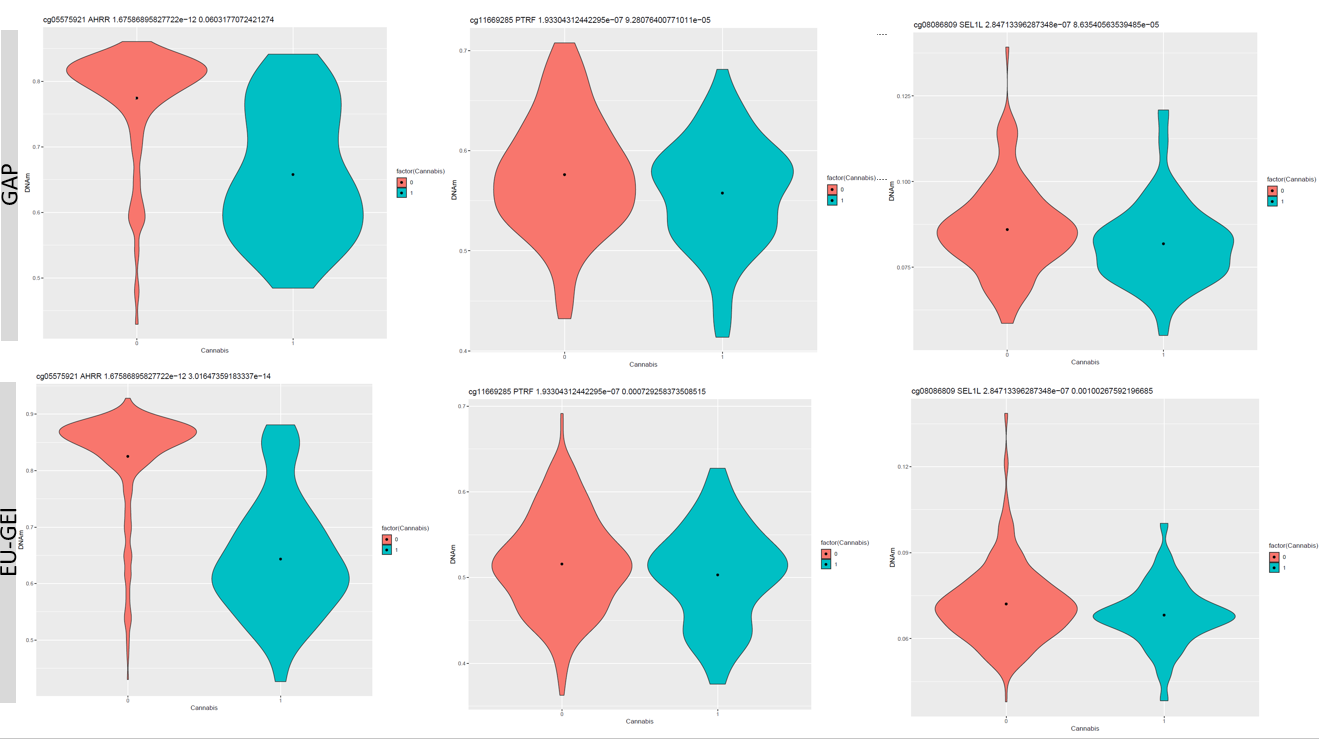


**Figure S5: DNA methylation beta values for the top probes in the current cannabis use meta-analysis in the two cohorts, the first number after the gene name is the meta-analysis p-value and the second is the p-value for the cohort-specific EWAS.** Current cannabis: 0= controls; 1=cannabis current user)


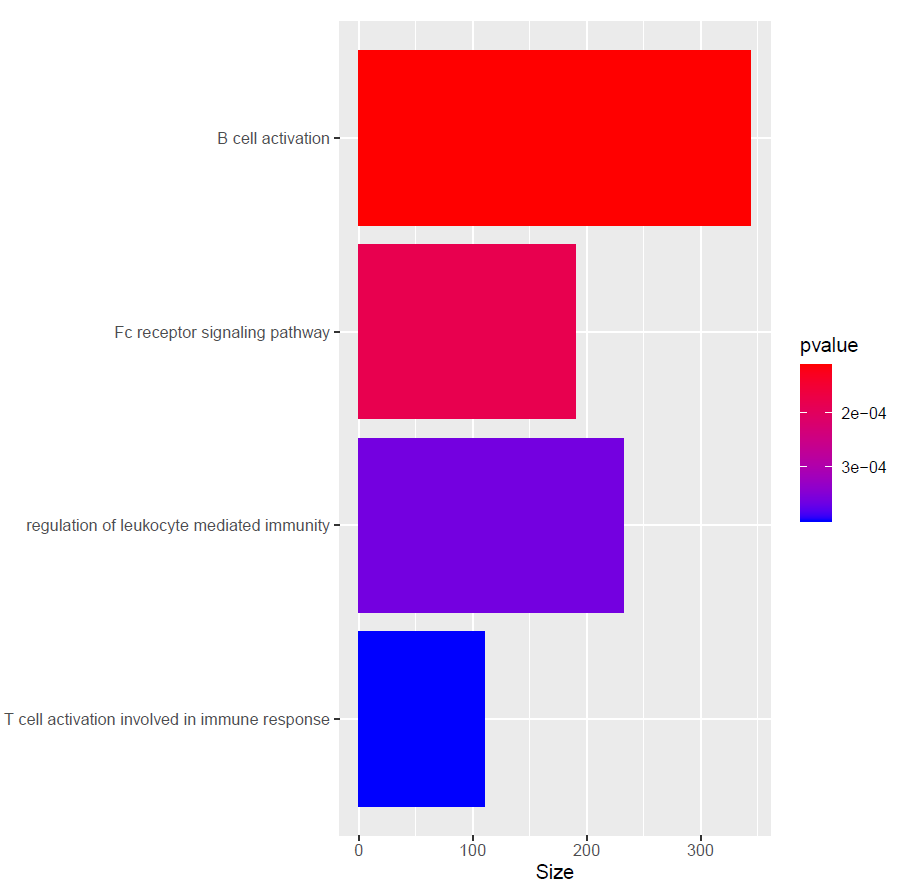


**Figure S6: Pathway analyses of current high potency cannabis users compared to never users failed to identify any pathways that survived multiple testing (padj >0.05**); however, the same pathways were identified at nominal significance as the main analysis.


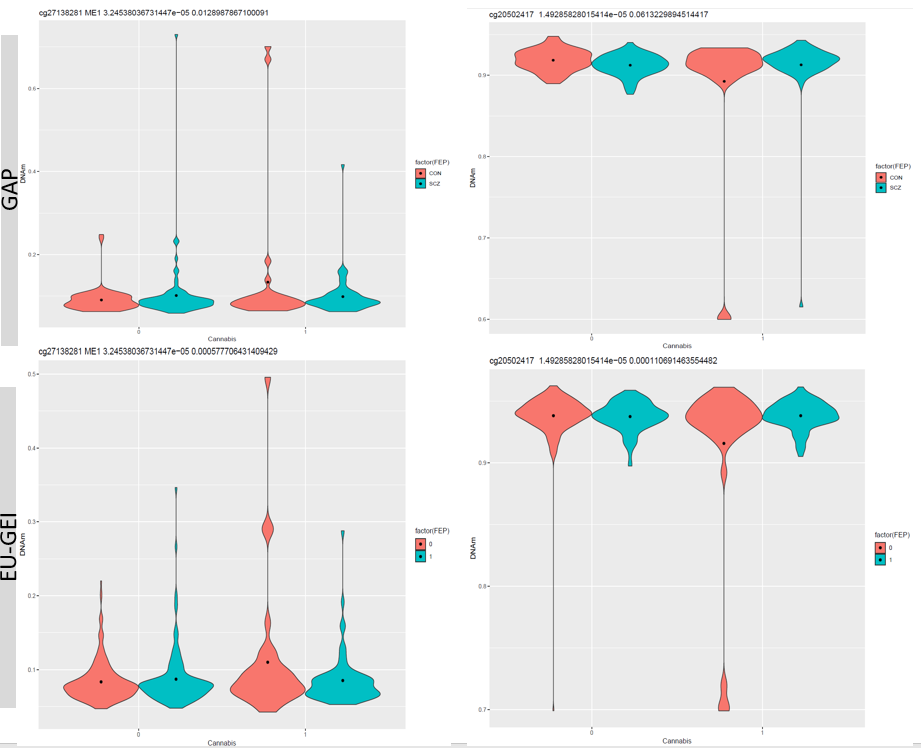


**Figure S7: DNA methylation beta values for the top probes in the current cannabis use interaction with psychosis meta-analysis in the two cohorts, the first number after the probe or gene name is the meta-analysis interaction p-value and then the second is the interaction p-value for the cohort-specific EWAS** ( FEP status: control = 0/con, FEP case = 1/SCZ: Current cannabis: 0= controls; 1=cannabis current user)


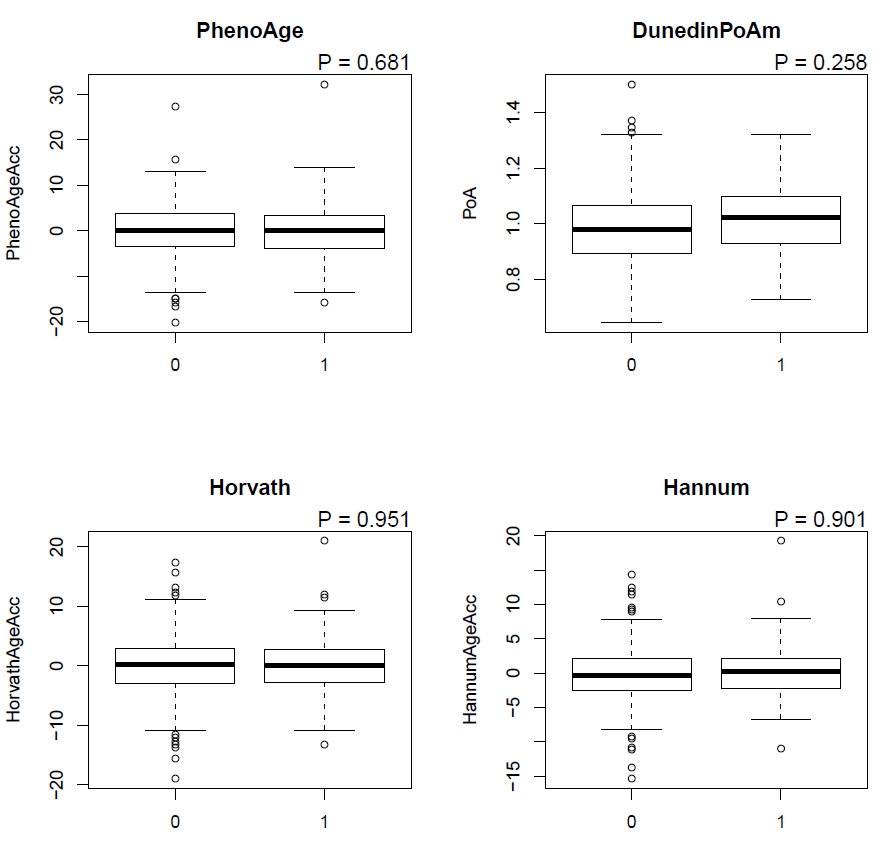


**Figure S8: DNA methylation Age acceleration calculated using four different epigenetic clocks in the EU-GEI cohort:** There was no evidence of age acceleration between current cannabis users and controls. (0= controls; 1=cannabis current user)


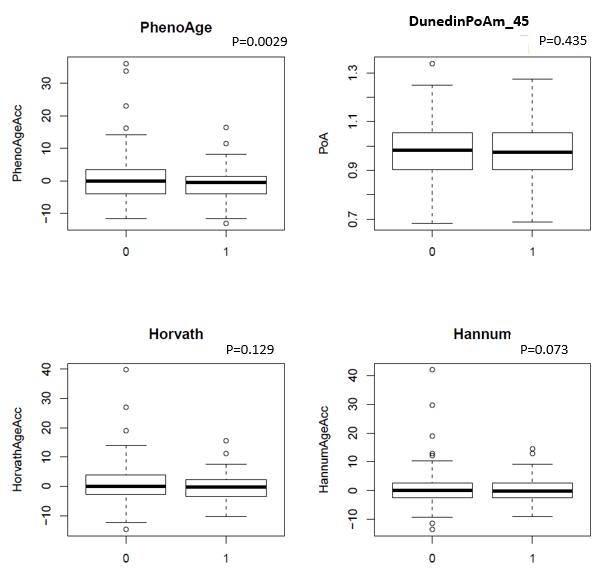


**Figure S9: DNA methylation Age acceleration calculated using four different epigenetic clocks in the GAP cohort:** There was no evidence of age acceleration between current cannabis users and controls. ( 0= controls 1=cannabis current user). PhenoAge showed a significant age deceleration (p= 0.003).
