# Supplementary material for: Methylomic signature of current cannabis use in two first-episode psychosis cohorts
Source: Mol Psychiatry. 2024 Oct 16;30(4):1277–86. doi: 10.1038/s41380-024-02689-0 (PMC11919776; doi:10.1038/s41380-024-02689-0)
Supplement: Supplementary file 2 — Supplementary Methods [file 41380_2024_2689_MOESM2_ESM.docx]

**Supplementary Methods:**

**Measurements**

*Cannabis Potency variable:* For both cohorts, participants were asked to report in their native language the type of cannabis they used and this was recorded in the Cannabis experience Questionnaire modified version (Di Forti et al Lancet Psychiatry 2015 and 2019) . For the EUGEI study the low-potency cannabis category (THC<10%) included hash/resin from UK and Italy, imported herbal cannabis from UK, Italy, Spain and France , Brazilian marijuana and hash and the Dutch Geimporteerde Wiet . The high-potency category (THC=>10%) included all the other types reported by the study participants in their original language street name such as: UK home-grown skunk/sensimilla, UK Super Skunk, Italian home-grown skunk/sensimilla, Italian Super Skunk, the Dutch Nederwiet, Nederhasj and geimporteerde hasj, the Spanish and French Hashis (from Morocco), Spanish home-grown sensimilla, French home-grown skunk/sensimilla/super-skunk and Brazilian skunk (Ref 1-9, as below) .

Accordingly, for the GAP study UK home-grown skunk/sensimilla, UK Super Skunk were included in the high potency category, while hash/resin and imported herbal cannabis from UK in the low potency category ^8^.

This classification of cannabis potency has been used in two separate epidemiological studies published in Lancet Psychiatry (Di Forti et al 2015 and Di Forti et al 2019). The first (Lancet Psych 2015) is based on the London based GAP sample and the second ( Lancet Psych 2019) is based on the EUGEI multicentre cohort and replicates the GAP study findings on the association between use of cannabis potency and ORs for psychotic disorders, supporting the validity of our potency measure. These references are available in the main text.

One of the strengths of our study is the multi-center structure. Despite differences in cannabis related drug policy between for instance in the UK and the Netherlands, in both London and Amsterdam we describe a similar distribution of self-reported prevalence of cannabis use in cases and controls.

To further support the validity of our “cannabis potency” variable, the data provided in the EMCDDA 2016 ^1^ represent the largest effort to describe variations in cannabis potency across Europe. They include as reliable as possible data such as the annual report provided by the Netherland government ^6^, which has a very detailed description of the THC and CBD concentration found in all the types of cannabis available in the Dutch coffee shops. As mentioned above, we added to the supplementary method section a detailed description of how we created the cannabis potency binary variable, using the actual name, in the original language, that participants (cases and controls) reported using.

We do know from both the Dutch potency estimates and from the UK potency data (ref reported above) that cannabis street types containing on average THC=>10% show consistently only low traces of CBD, the latter more likely to be present in the types of cannabis with modest to low THC %. It is well known that the cannabis sativa plant is genetically programmed to produce either high THC or high CBD but not high concentration of both ^10^. Therefore, as we had only reliable data on CBD % from two of the five countries (Netherlands and UK), we chose to rely on THC % alone and avoid a guessing exercise on CBD %.

1. European Monitoring Centre for Drugs and Drug Addiction (EMCDDA). European Drug Report 2016: Trends and Developments. Publications Office of the European Union. Luxembourg 2016.
2. European Monitoring Centre for Drugs and Drug Addiction (EMCDDA), Ministry of Health and Consumer Affairs (Spain). Spain National Report to the EMCDDA 2012.
3. Niesink R RS. THC-concentraties in wiet, nederwiet en hasj in Nederlandse coffeeshops (2012-2013). AF1221. Utrecht: Trimbos-instituut; 2013.
4. Observatoire Français des Drogues et des Toxicomanies (OFDT). Drogues, chiffres clés. Paris2015.
5. Zamengo L, Frison G, Bettin C, Sciarrone R. Cannabis potency in the Venice area (Italy): Update 2013. Drug Testing and Analysis. 2015;7(3):255-8.
6. Niesink RJM, Rigter S, Koeter MW, Brunt TM. Potency trends of Δ9‐tetrahydrocannabinol, cannabidiol and cannabinol in cannabis in the Netherlands: 2005–15. Addiction. 2015;110(12):1941-50.
7. de Oliveira GL, Voloch MH, Sztulman GB, Neto ON, Yonamine M. Cannabinoid contents in cannabis products seized in São Paulo, Brazil, 2006–2007. Forensic Toxicology. 2008;26(1):31-5.
8. Potter DJ, Clark P, Brown MB. Potency of Δ9–THC and Other Cannabinoids in Cannabis in England in 2005: Implications for Psychoactivity and Pharmacology*. Journal of Forensic Sciences. 2008;53(1):90-4.
9. Hardwick S KS. Home Office Cannabis Potency Study 2008. London: Home Office Scientific Development Branch; 2008.
10. Potter DJ, Hammond K, Tuffnell S, Walker C, Forti MD. Potency of Δ9–tetrahydrocannabinol and other cannabinoids in cannabis in England in 2016: Implications for public health and pharmacology. Drug Testing and Analysis. 2018;10(4):628-35.
